# Impact of Steam-Exploded Feather Incorporation on the Biodegradation Performance of Renewable Biocomposites

**DOI:** 10.3390/polym17070910

**Published:** 2025-03-28

**Authors:** Julen Vadillo, Sarah Montes, Hans-Jürgen Grande, Eveline Beeckman, Steven Verstichel, Jonna Almqvist

**Affiliations:** 1 CIDETEC, Basque Research and Technology Alliance (BRTA), Paseo Miramón, 196, 20014 Donostia-San Sebastian, Spain; jvadillo@cidetec.es (J.V.); hgrande@cidetec.es (H.-J.G.); 2 Advanced Polymers and Materials: Physics, Chemistry and Technology Department, University of the Basque Country (UPV/EHU), Avda. Tolosa 72, 20018 Donostia-San Sebastian, Spain; 3 Normec OWS, Pantserschipstraat 163, 9000 Gent, Belgium; eveline.beeckman@normecgroup.com (E.B.); steven.verstichel@normecgroup.com (S.V.); 4 Department of Biorefinery and Energy, RISE Research Institutes of Sweden, S-892 50 Örnsköldsvik, Sweden; jonna.almqvist@ri.se

**Keywords:** chicken feather, biodegradable polymers, biocomposites, agricultural bioplastic, waste management

## Abstract

The increasing environmental concerns regarding plastic waste, especially in agriculture, have driven the search for sustainable alternatives. Agricultural plastics, such as mulching films and greenhouse covers, are heavily reliant on petrochemical-derived materials, which persist in the environment and contribute to long-term pollution. This study explores the use of biodegradable biocomposites made from steam explosion-treated chicken feathers and various polymer matrices to address these issues. Chicken feathers, a waste by-product of the poultry industry, present an excellent biodegradability as a result of the steam explosion treatment and contain nitrogen, potentially enhancing soil fertility. The biocomposites were characterized by thermal stability, mechanical properties, and biodegradability, and ecotoxicity assessments were carried out studying the incorporation of feathers into the soil. Results showed that the incorporation of treated chicken feathers increased the water absorption capacity of the composites, promoting faster disintegration and biodegradation. In particular, biocomposites made with polyhydroxyalkanoates and Poly(3-hydroxybutyrate-co-3-hydroxyvalerate) exhibited a significant increase in degradation rates, from 3–10% in the first month for pure matrices to 40–50% when reinforced with treated feathers. Meanwhile, those made from polylactic acid showed slower degradation. Furthermore, the addition of feathers positively influenced crop growth at low concentrations, acting as a slow-release fertilizer. However, high concentrations of feathers negatively affect plant growth due to excess nitrogen. These findings highlight the potential of poultry feathers as a valuable, sustainable filler for agricultural bioplastics, contributing to waste valorization and environmentally friendly farming practices.

## 1. Introduction

The increasing environmental concerns related to plastic waste have prompted a global shift towards the development of more sustainable alternatives. Agriculture is one of the sectors heavily reliant on plastics, with extensive use in applications such as mulching films, greenhouse covers, and seed trays [1]. The demand for agricultural plastics was at about 12.5 Mt in 2019 [2], and it is expected to increase due to the rising demand for food and to cover the rapidly increasing population needs [3]. Conventional plastic mulch films are typically composed of low-density such as polyethylene (PE), polyvinylchloride (PVC) or ethylene-vinyl acetate (EVA) copolymers [4], but these plastics, which are derived from petrochemicals, represent a significant environmental issue due to their persistence in the environment and challenges in disposal and recycling [5]. Additionally, these agricultural plastics often accumulate in soils, degrading soil health, and contributing to long-term pollution [6]. Hence, there is an urgent need to develop biodegradable materials that not only meet the functional requirements of agricultural applications but also reduce environmental impact and associated disposal costs.

Biodegradable plastics offer a promising solution to environmental issues associated with plastic use, allowing a reduction in plastic waste and potentially decreasing the costs linked to the recovery and disposal of materials at the end of their lifecycle [7]. The production of such materials requires renewable and low-cost feedstocks, preferably from waste sources, to ensure both economic and environmental sustainability. In this context, the valorization of industrial biodegradable waste products, such as poultry feathers, emerges as a viable approach to creating biodegradable materials while simultaneously addressing waste management issues. Poultry feathers are a by-product of the poultry industry, which generates an estimated 40 million tons of feathers annually [8], most of which are either incinerated [9], landfilled [10], or used in low-value applications [11]. Given their availability and their biodegradability, their incorporation into agricultural bioplastics seems to be a potential valorization method for creating value-added products.

Chicken feathers are composed predominantly of keratin, a fibrous protein that provides feathers with a combination of light weight, high strength, and thermal and acoustic insulation properties [12]. Additionally, keratin is rich in nitrogen, a key nutrient for plants, meaning that when these biodegradable materials degrade in the soil, they can potentially act as a slow-release fertilizer, further enhancing the sustainability of their use in agriculture [13]. The incorporation of chicken feathers into polymer matrices has been the subject of increasing interest in recent years. Previous studies have explored the use of feather fibers in different polymeric matrices, such as polypropylene [14,15], polyethylene [16], and polylactic acid [17], with the aim of developing materials with improved properties. Mishra et al. reported an increase in the Young modulus of PP composites, with the composite-containing feathers exhibiting a 1.87% of increase compared to neat PP. However, this enhancement in stiffness was accompanied by a reduction in tensile strength, with an 8% decrease [15]. The same tendency was observed in a study carried out by Baba et al., where the incorporation of chicken feather fibers into PLA resulted in a 5.4% increase in tensile modulus while reducing tensile strength and elongation at break compared to pure PLA [17]. Most of the literature is focused on the properties of the obtained composites; however, only a few authors studied the influence of the incorporation of chicken feathers on the biodegradation behaviour of the obtained biocomposites [18], and no research is reported about using steam explosion-treated feathers for agricultural biodegradable plastic fabrication.

Steam explosion (SE) is a well-known treatment for fractionating wood, and to a lesser extent biomass, in which the starting material is treated with hot steam and under pressure, followed by an explosive decompression that results in a rupture of the structure of the material [19]. In a previous work, the effectiveness of this treatment for enhancing the biodegradability and biodegradation rate of the chicken feathers was demonstrated [20], so it is expected that its incorporation into different polymer matrices will modify in a greater way their disintegration and biodegradation behaviour. In addition to this, the use of keratin of feathers, a high nitrogen content source, offers a path toward producing materials that not only degrade naturally in the environment but also contribute nutrients to the soil, reducing the need for additional external fertilization.

This work studies the preparation and characterization of biocomposites composed of chicken feathers treated by SE and different polymeric matrices. The prepared biocomposites have been characterized in terms of thermal, morphological, and mechanical properties, and their biodegradability, disintegration behaviour, and ecotoxicity have been tested and assessed in soil conditions, providing insights into their suitability for agricultural applications. By focusing on the valorization of poultry feathers, this work not only seeks to address a waste management challenge but also contributes to the broader effort of developing sustainable materials for agriculture, supporting the transition towards more circular and eco-friendly farming practices.

## 2. Experimental Section

### 2.1. Materials

For the preparation of the biocomposites containing chicken feathers, the following biodegradable matrices were selected and provided by Bio-Mi Ltd. (Matulji, Croatia): polylactic acid (PLA) (Luminy LX175 from Corbion (Amsterdam, The Netherlands)), polybutylene adipate terephthalate (PBAT) (Ecoworld from Jinhui (Taiyuan, China)), poly(3-hydroxybutyrate-co-3-hydroxyvalerate) (PHBV) (Enmat Y1000PXX from TianAn (Beilun, China), and polyhydroxyalkanoate (PHB) (Enmat Y3000P from TianAn. Sterilized raw chicken feathers were provided by Cedrob S.A (Ciechanów, Poland). Feathers were soaked and washed with detergent for 10 min (Dehaclin Fn 100 from CHT (Tübingen, Germany)) and were posteriorly dried and sterilized (40 min; T = 120 °C; P = 2 bar).

### 2.2. Steam Explosion Treatment of Chicken Feathers

The steam explosion treatment of feathers was carried out in a 40 L reactor designed for up to 28 bar and was heated with direct steam. Feathers were introduced into the steam explosion reactor and treated at 190 °C for 4 min. The obtained steam explosion-treated feathers (SEFs) were dried at 95 °C until a constant weight of the samples was obtained to remove the remaining moisture and subsequently ground to obtain particles of 0.5 mm.

### 2.3. Preparation of Biocomposites

The biocomposites containing SEFs were manufactured by using a twin-screw extruder Process 11 from Thermo scientific (Bordeaux, France). In Table 1, the composition and processing conditions of the prepared formulations are displayed.

The obtained compounds were fed again into the twin screw extruder equipped with a heated flat head to obtain a uniform ribbon with a controlled thickness in the range of 0.4–0.5 mm. These ribbons were used for the disintegration testing of the biocomposites.

Additionally, for the mechanical test, specimens were produced by injection moulding. For this purpose, the different compounds were injected into dog-shaped specimens following the ASTM D638 (type IV) [21] using a Babyplast injector from Cronoplast (Barcelona, Spain).

### 2.4. Characterization

#### 2.4.1. Density

The density of the biocomposites was determined experimentally according to ISO 9427 [22]. Three rectangular samples of each composite with a known volume were weighed, and the density was determined as the ratio of the mass to volume. The average and standard deviation were reported.

#### 2.4.2. Thermogravimetry (TGA)

The thermal stability was measured by thermogravimetric analysis using a TGAQ500 (TA Instruments, New Castle, PA, USA). Dynamic measurements were performed from 25 to 800 °C at a heating rate of 10 °C min^−1^ using a constant nitrogen flow of 60 mL min^−1^ to prevent thermal oxidation processes of the sample.

#### 2.4.3. Field-Emission Scanning Electron Microscopy (FE-SEM)

The interface of the prepared biocomposites before and during the biodegradation test was analyzed by field-emission scanning electron microscopy. The microphotographs were taken with a Carl Zeiss Ultra Plus field-emission scanning electron microscope (FE–SEM, Oberkochen, Germany) equipped with an energy-dispersive X-ray spectrometer (EDXS). For the FE–SEM analysis, samples were previously coated with Au.

#### 2.4.4. Water Absorption of Biocomposites

Water absorption of the biocomposites was determined by immersion of the specimens vertically in distilled water at 25 °C for 24 h (ASTM D570-98 [23]). First, rectangular specimens (24 × 12 × 2.1 mm^3^) were cut from tensile testing fracture specimens and air-dried at 60 °C for 24 h, cooled in a desiccator and weighed (conditioned weight). Then, samples were soaked in water for 24 h and wiped with paper to remove the excess of water on the surface of the specimens before weighing (wet weight) at fixed time intervals. Three specimens were tested with an analytical balance of 0.1 mg precision, and the average and standard deviation were reported. The percentage of water absorption (WA in %) was calculated using Equation (1):

(1) $$WA\left(\%\right)=\frac{W_{w}-W_{d}}{W_{d}}\times 100$$

where WA is the water absorption percentage, W_w_ is the weight of the sample after the immersion in water, and W_d_ is the original weight of the sample before the test.

#### 2.4.5. Mechanical Testing

The mechanical properties of the biocomposites (Young’s modulus, tensile strength, and elongation at break) were evaluated using a tensile test according to the ISO 527 standard [24] with a universal testing machine model 3365 Instron (Norwood, MA, USA) and controlled by Bluehill 3 software developed also by Instron (Norwood, USA). The initial length of the test specimens was 25.4 mm, and a crosshead speed of 10 mm min^−1^ was used. The number of specimens tested for mechanical properties was 5 for average calculations.

#### 2.4.6. Biodegradation in Soil

The standard soil biodegradation test of the biocomposites was performed according to ISO 17556 [25]. The reference item cellulose and the test items were added as powder, directly mixed with standard soil, and incubated in the dark at an ambient room temperature (25 °C ± 2 °C). Biodegradation took place through microbial activity, and as a result, carbon dioxide and water were produced. The CO_2_ was captured in KOH, and the CO_2_ production was regularly determined by titration, which allowed us to calculate the cumulative CO_2_ production. The percentage of biodegradation could be calculated as the percentage of solid carbon of the test item, which was converted into gaseous, mineral C under the form of CO_2_. A test item demonstrated a satisfactory level of biodegradation when 90% absolute or relative biodegradation was reached. The maximum allowed test duration determined by this standard is two years.

#### 2.4.7. Disintegration in Soil

The disintegration of the test items was qualitatively evaluated during an incubation period in soil up to 48 weeks. The standard soil inoculum consisted of a mixture of 70% industrial quartz sand, 10% kaolinite clay, 16% natural soil, and 4% mature compost (percentages expressed on dry weight basis). Before its use, the natural soil was sieved on a screen of 2 mm, while the mature compost was sieved on a screen of 5 mm. The fine fractions were used for the inoculum. Finally, salts were added to the standard soil mixture by means of nutrients solution. The soil inoculum was maintained with a water content between 40% and 60% of the total water holding capacity and a pH in the range of 6.0–8.0. The test items were cut into 2.5 cm × 2.5 cm pieces, mixed with soil inoculum and incubated at 25 °C ± 2 °C in the dark. The soil was stirred and moistened regularly (every 2 weeks), if needed, in order to guarantee optimal test conditions. At the same time, the disintegration of the test materials was monitored.

#### 2.4.8. Ecotoxicity Test in Soil

The test is executed in line with the OECD 208 Terrestrial Plant Test: Seedling Emergence and Seedling Growth Test [26] with the following procedure. The test was performed in flowerpots of 500 mL, containing blank soil or test soil. Each soil was tested in 4 replicates. At the start of the test, each flowerpot was filled with 300 g of soil. Subsequently, 100 seeds of cress or barley were put on top of the soil and covered with a thin layer of siliceous sand. Finally, an extra amount of demineralized water was added to assure optimal moisture content. After the flowerpots were completely prepared, they were covered with a glass plate and incubated at a constant temperature of 20 °C ± 2 °C in the dark. After germination, the plate was removed, and the pots were exposed to a light intensity of at least 3000 lux for at least 12 h per day. The test was finished 14 days (±2 days) after 50% of the control seedlings had emerged. At the end of the test, the total fresh and dry weight of the above-soil plant material was determined for each flowerpot separately. Also, the germination rate was measured. The toxicity of possible residuals of the test item was evaluated by comparing the results on germination and plant yield of test soil to blank soil.

The pH as well as the ammonium–nitrogen (NH_4_^+^-N) and nitrate/nitrite–nitrogen (NO_x_—N) content were analyzed in the studied soils. Both ammonium-N and nitrate/nitrite–nitrogen contents were determined by spectrophotometric detection at 660 nm and 540 nm, respectively. Finally, the pH was determined by using a pH meter and diluting the sample with distilled water at a ratio of 5 to 1.

## 3. Results and Discussion

In Table 2, the density of neat polymers and corresponding biocomposites containing treated feathers is shown. The incorporation of the SEFs resulted in a decrease in the relative density of the biocomposite in all formulations. This behaviour can be explained due to the lower density of the feathers compared with the matrices, as observed previously in the literature for raw feathers [27]. Considering that the SEFs presented a higher apparent density than treated feathers [20], the decrease in the density of the biocomposites is less remarkable. Additionally, the adhesion between the particle and the matrix can also affect the density, resulting in the apparition of voids and hence a decrease in the density [28,29].

The water absorption capacity of the biocomposites was determined by means of swelling test. The results are displayed in Table 2, whereas the obtained curves are shown in Figure S1. In general, the incorporation of treated feathers in the polymer backbone led to a clear increase in the water absorption capacity, whereas the neat matrices showed a low absorption capacity due to their hydrophobic nature. This increase in the water absorption capacity for biocomposites containing feathers was previously reported in the literature for blends containing untreated feathers and PLA [18], and it was justified due to the hydrophilic nature of feathers [30,31]. Moreover, it is expected that the incorporation of hydrophilic fillers into a hydrophobic matrix could cause poor compatibility in the interface. This will result in the apparitions of voids, which can enhance even more the water absorption capacity. In general, most of the biocomposites showed a similar increase in the swelling capacity as a result of the feather incorporation, with the exception of PLA, in which a slighter effect was observed. This behaviour could be attributed to the higher hydrophobicity of the PLA compared with the other matrices [32,33].

**Figure 1:**
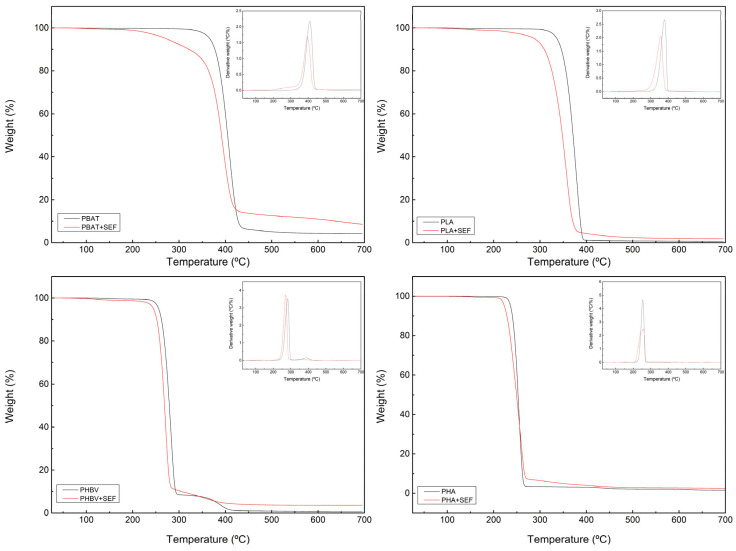
Degradation curves of the prepared formulations with and without treated feathers.

The thermal stability of the matrices and the prepared biocomposites have been tested by TGA. Figure 1 showed the thermal decomposition of neat polymers and biocomposites with SEF. In the case of the neat polymers, all matrices exhibited different degradation curves depending on their nature, with PBAT and PLA showing a greater thermal stability compared with PHB and PHBV [34].

The starting degradation temperature of all biocomposites with feathers is slightly lower than their corresponding neat matrices. All biocomposites are thermally stable until 230–240 °C; above this temperature, biocomposites start degrading probably due to the presence of treated feathers, as are reported to start degrading around 200 °C according to a previous study [20]. For temperatures above 400 °C, all the polymer matrices were completely decomposed, presenting a residue which can be attributed to the ash of the chicken feathers.

**Figure 2:**
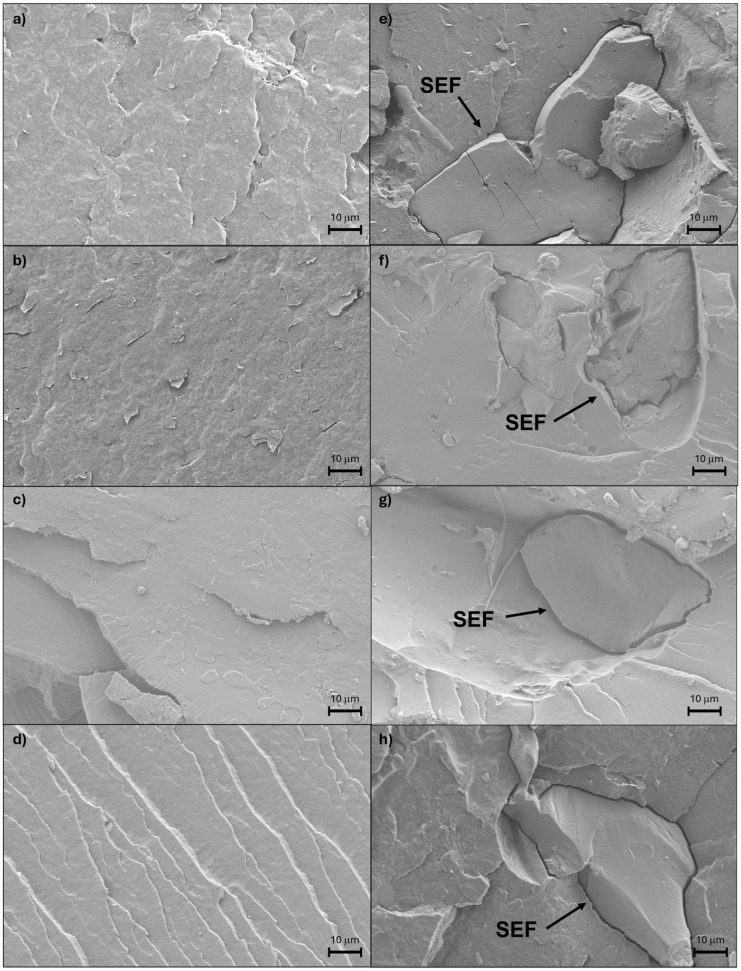
FE-SEM images of the prepared matrices and biocomposites containing SEF (**a**) PHB, (**b**) PHBV, (**c**) PLA, (**d**) PBAT, (**e**) PHB + SEF, (**f**) PHBV + SEF, (**g**) PLA + SEF, and (**h**) PBAT + SEF.

The interface between SEF and the different matrices has been studied by FE-SEM. In Figure 2, the images of biocomposite formulations with treated feathers are displayed. In all cases, the interface between the polymer matrix and the treated feathers showed a clear gap surrounding the filler. This fact suggested a poor compatibility between the hydrophilic feather and the hydrophobic polymeric matrix [35], which could lead to a decrease in the mechanical properties. However, this gap produced due to the low compatibility between the treated chicken feathers and the polymer matrix led also to an increase in the moisture absorption capacity, as observed previously, which posteriorly will help with the degradation of the biocomposites. Comparing the different formulations, the aforementioned gap in the interface seems to be similar in all cases, confirming that all matrices showed a low compatibility with SEF. The SE treatment did not seem to have a significant effect on the compatibility between the matrix and the treated feathers. In previous works, untreated chicken feathers exhibited similar compatibility problems [36].

The mechanical properties of biocomposites were tested by a tensile test in order to determine the influence of the incorporation of the treated feathers. The results, which are displayed in Table 3, showed a general decrease in the mechanical properties when feathers were added. In all cases, an increase in the young modulus was observed when SEFs were incorporated in comparison with the pristine materials but also a decrease in the tensile strength and strain at break. This decrease in the tensile strength is produced as a consequence of the low mechanical properties of chicken feathers, which in this case play the role of a filler rather than a reinforcement. Additionally, other reasons, such as the aforementioned low compatibility between feathers and the different matrices [36], which resulted in the apparition of a gap between the matrix and the SEF particle, could also explain the fragilization of the biocomposites in comparison with the pristine materials.

Similar results were obtained by other authors, in this case for the incorporation of raw chicken feathers in polymeric matrices. Chen et al. demonstrated that even if a low amount of feathers resulted in a slight increase in the mechanical properties, the incorporation of percentages above 5 wt% led to a clear decrease in mechanical properties in chicken feather/PLA biocomposites [37]. Aranberri et al. also obtained a decrease in the tensile strength, incorporating up to 60 w t% of feathers into a PBAT-based commercial polymer [18]. In the present work, the negative effect of the incorporation of feathers treated on tensile strength is more of an issue in PLA and PBAT than in the case of PHB and PHBV. This can be explained owing to the different compatibility of the treated feathers with the different polymers used as well as due to the inherent mechanical properties of each material.

**Figure 3:**
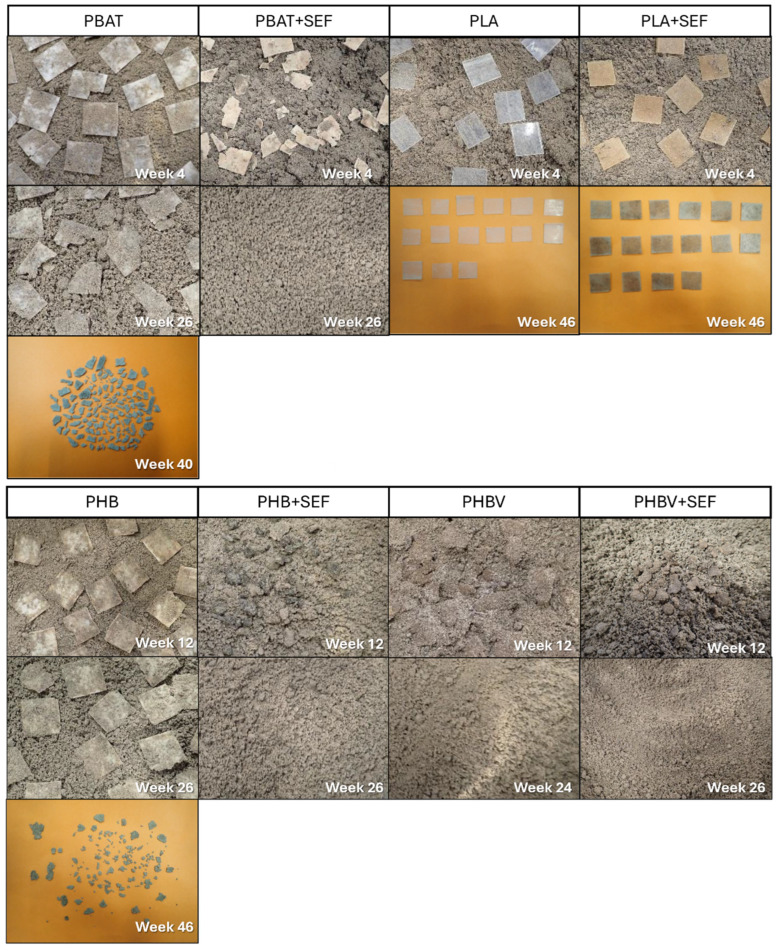
Images of the disintegration process of the different prepared bioplastic at different stages.

The disintegration process of the prepared biocomposites was studied in order to determine the effect of the incorporation of treated feathers in the decomposition of the material in the soil. This test will give a greater insight into how bioplastics will behave in a real environment and hence evaluate their potential use in agriculture. In Table 4, the results obtained for the different materials are displayed up to 46 weeks of testing, whereas, in Figure 3, images of the disintegration process of the different tested materials are shown.

The incorporation of SEF in the different polymeric matrices leads to an increase in the disintegration rate in both biocomposites containing PBAT and PHB, falling apart earlier in comparison with the ones without feathers. In all cases, with the exception of the PLA, the samples containing SEF completed the disintegration test in less than 46 weeks. These results agree with the behaviour observed for other types of organic fillers reported in the literature such as Starch in PLA [38] or lignin in polyvinyl alcohol (PVA) [39], where the incorporation of organic particles accelerated the disintegration of the biocomposites. Generally, the incorporation of hydrophilic fillers in a hydrophobic matrix led to an increase in the water absorption capacity, as was previously observed in Table 2. This increase in the water absorption is produced in the hydrophilic particles which experienced a swelling. As a result of internal tensions between the swollen filler and the matrix, small cracks appear in the interface. The material becomes more brittle as these cracks become bigger until the material is disintegrated into smaller fragments [40]; this process accelerates the disintegration of the biocomposites in comparison with the pristine materials. In addition to this, the particles act as channels which are more accessible to the water and the microorganisms, not only accelerating the disintegration, but also the biodegradation [41].

Additionally, the gap in the interface between the matrix and the feathers observed in the FE-SEM due to the bad compatibility between the hydrophilic filler and the hydrophobic matrix eases even more the water uptake of the material, accelerating the aforementioned process where the material becomes more brittle due to the humidity, resulting in the fragmentation of the bioplastic.

Comparing the different matrices, the effect of the SEFs’ incorporation seems to be more effective in polymers with lower disintegration rates (PHB, PBAT). In fast disintegrating polymers such as PHBV, the effect seems to be the opposite, requiring slightly more time to disintegrate the biocomposite than the matrix. The non-positive effect of the incorporation of organic fillers into a PHBV matrix as far as disintegration is concerned was previously reported in the literature [42]. However, the comparison of samples containing the same matrix but different organic fillers is in most cases not very accurate and is difficult to make correlations since many parameters have to be considered such as chemical composition, particle size or structure, among others. It has to be considered also that the PHBV itself presents a very fast disintegration and biodegradation, and in this case, the inclusion of the feathers can interfere slightly with the proper disintegration process of the PHBV. In the case of the PLA, despite the positive effects that can be seen in the samples where the SEFs are incorporated, the material is almost intact after 46 weeks of tests. As reported by other authors, PLA requires temperatures above its glass transition temperature (55–60 °C) to be properly disintegrated and biodegraded [43]. Indeed, its disintegration is very slow, and it can take from several months to years to fully disintegrate [44], so in this case, the incorporation of the feathers did not improve the disintegration significantly.

To complete this study, FE-SEM images were taken from the tested ribbons in order to visualize the disintegration process over time. In Figure S2, the images of all composites, with the exception of PLA + SEF, at different checkpoints are displayed. As can be observed, in the case of materials containing SEF, the apparition of cracks can be seen in earlier stages in comparison to the matrices, confirming the effect of the chicken feathers as an accelerator of the disintegration.

**Figure 4:**
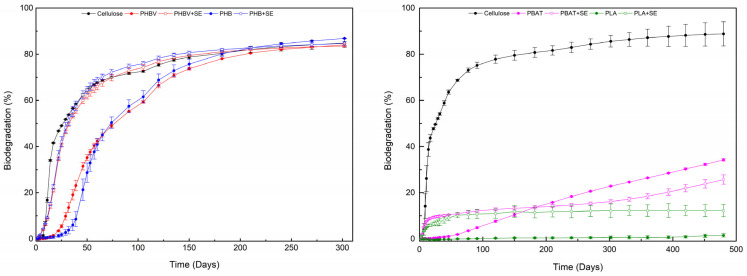
Biodegradation curves of the prepared bioplastic: PHBV and PHB series (**left**) and PLA and PBAT series (**right**).

The biodegradation in soil conditions has also been studied to determine the effect of feathers treated by steam explosion on the biodegradation behaviour of biocomposites. The results, which are displayed in Figure 4 and in Table S1, showed a different behaviour depending on the polymeric matrix used. Materials containing PBAT showed a continuous biodegradation up to 300 days; however, the relative biodegradation seems to be very slow with and without feathers. In PLA compounds, the biodegradation of the pure matrix is residual, whereas in the biocomposite-containing treated feathers, 10% of biodegradation is observed, corresponding to the proper biodegradation of the added feathers. On the contrary, biocomposites containing PHB and PHBV with and without treated feathers presented a fast biodegradation, obtaining a complete relative biodegradation of the biocomposites in less than 200 days.

Biocomposites containing PHB and PHBV with and without treated feathers presented a fast biodegradation, obtaining a complete relative biodegradation of the bioplastic in less than 200 days. In PHB and PHBV, the incorporation of feathers seems to affect the biodegradation behaviour of the biocomposite. Thus, despite obtaining a final similar biodegradation percentage, the addition of SEFs has an accelerating effect. In fact, after one month of testing, both PHB and PHBV materials showed a biodegradation percentage between 3 and 10%, whereas the same systems with SE feathers presented a higher percentage in the range of 40–50%. This positive effect of the SEFs in the initial steps of the biodegradation can be explained due to the extremely fast biodegradation of the proper feathers as a result of the steam explosion treatment reported in a previous study [20], which also led to the apparition of more accessible sites to the microorganisms. The acceleration effect produced in the biodegradation of biocomposites owing to the incorporation of organic fillers has been previously reported in the literature in the case of natural fibers reinforcing PHB [45]. For PBAT and PLA systems, the accelerating effect of the feathers’ incorporation was also observed in the first month of tests. In PLA compounds, the biodegradation of the pure matrix is residual, whereas in the biocomposite-containing treated feathers, 10% of biodegradation is observed, corresponding to the proper biodegradation of the added feathers. The PBAT sample with SEF quickly reached 10% biodegradation, while the pure PBAT sample only started to degrade after a lag phase of approximately 60 days. However, the biodegradation rate of the PBAT sample with SE feathers slowed down, resulting in a higher biodegradation level for the pure PBAT matrix from 180 days onwards. The biodegradation rate of the PBAT sample with SE feathers gradually started to pick up again, and both samples continued degrading at similar rates. It can be assumed that the intermediate lag phase of the PBAT sample with SEFs results from the adaptation phase of the microbial community from SEFs as a main food source to the PBAT matrix. Since feathers are made of keratin, many bacteria and fungi have evolved to degrade using keratinase enzymes, thus making it easily biodegradable. In contrast, PBAT is a synthetic polymer, which requires specific microbial adaptation and enzymatic processes for breakdown (also explaining the lag phase for pure PBAT). Therefore, the microorganisms will preference the easily biodegradable feathers, and once this source is depleted, they will shift to degrading the complex polymer chains in PBAT.

Ecotoxicity tests were carried out in order to analyze the effect of the chicken feathers on the plant growth once the biocomposite is disintegrated. For that reason, steam explosion-treated feathers (SEFs) and untreated feathers (RFs) were mixed with soil in different concentrations (1, 0.1, and 0.05 wt%, denoted as S1, S0.1, and S0.05, respectively), and the effect on the crop of cress and barley was studied. It is expected that the percentage of feathers released to the soil after the disintegration of the biocomposite will be low, so probably a lower amount of feathers will be the most representative sample to predict the effect of the bioplastic containing feathers. Table 5 shows the germination rate and weight yield of both types of crops in comparison with a soil with and without nutrients, as well as the nitrogen content of the soil containing different amounts of feathers.

As was expected, the presence of higher amounts of feathers in soil resulted in a higher nitrogen content, since feathers are rich in this element [46]. Regarding the comparison between untreated and treated feathers, no differences were observed in the case of low amounts of feathers. However, for 1 wt%, an increase in the nitrogen content was measured in SEFs compared to RFs. In this case, the faster biodegradation of SEFs led to a faster release of nitrogen, resulting in a higher concentration in the soil.

Analyzing the results of the crop growth, the incorporation of both treated and untreated feathers in high concentrations seems to have a negative effect in the germination rate in both barley and cress crops. This effect was reported previously by other authors for different types of crops such as watermelon and cucumber [47], bananas [48] or sugarcane [49], among others. The presence of high amounts of ammonium in soil can affect the cell viability and inhibit root growth [50] but can also cause ionic imbalances and acidification, negatively affecting the survival and growth of the crop [51]. For the low amount of feathers, the germination rate was similar to the observed ones for blank soils with and without nutrients in the case of barley. However, for cress crops, a positive effect was measured as far as germination rate is concerned, obtaining higher rates than the soil without nutrients and similar to the soil with nutrients. In this case, the presence of feathers in the soil acts as a fertilizer, improving the growth of the crop for low amounts of nitrogen [52].

Regarding the yield of the crops, the negative effects of the high amounts of nitrogen content in soil were observed in both crop types. In contrast, as happened with the germination, with lower amount of feathers (both treated and untreated feathers), the effect is positive, obtaining slightly higher yields of both crops than the blank soil without nutrients. As described by other authors, the incorporation of organic materials, which release nitrogen slowly, can improve soil fertility and minimize nitrogen loss during crop growth, thereby enhancing nutrient uptake and overall soil health [53].

**Figure 5:**
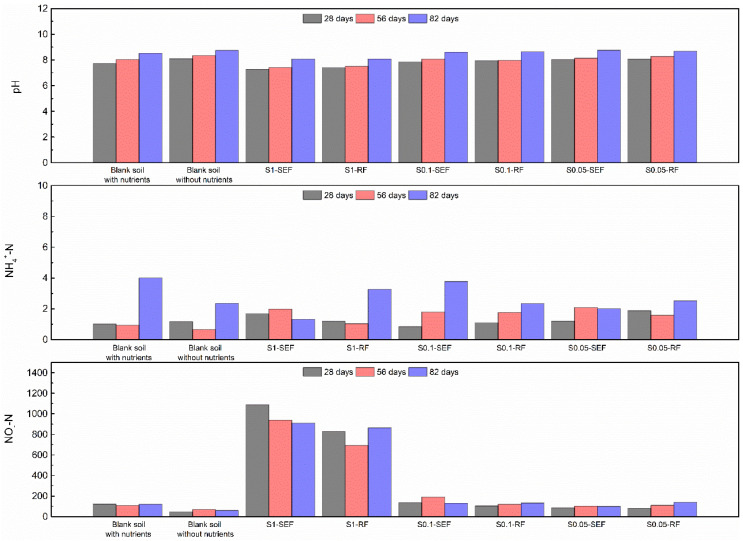
pH, NH_4_^+^-N, and NO_x_-N analysis of soils with different feather content.

To complete the ecotoxicity assessment, during the incubation period in soil, in all series, the pH, NH_4_^+^-N, and NO_x_-N were analyzed on a monthly basis. These parameters were studied due to their crucial role in understanding soil health and its capacity to support life. The pH of the soil is a key indicator of chemical conditions that affect nutrient availability and microbial activity, whereas NH_4_^+^-N (ammonium nitrogen) and NO_x_-N (nitrate and nitrite hydrogen) are essential for evaluating nitrogen cycling, which is crucial for plant growth and microbial processes [54]. The results are shown in Figure 5.

The pH slightly increased during the incubation period for all soil series. After 82 days of incubation, the pH varied between 8.1 and 8.8. This increase in the pH in all cases is produced due to the incorporation of nitrogen sources, which tend to have a basic effect on soil. Nitrate ions can combine with basic cations like calcium, magnesium, and potassium, reducing the concentration of hydrogen ions in the soil solution and thus increasing pH [55].

Low ammonium levels were observed in most of the soils during the initial incubation period, not observing any remarkable differences comparing treated and untreated feathers, as well as feather content in the soil. In some cases, however, the ammonium level experienced an increase after 82 days, as is observed for the reference blank soil with and without nutrients. During microbial activity, organic nitrogen from feathers is converted into inorganic forms like ammonium through the process of mineralization gradually increasing ammonium levels over time [56,57].

Regarding the nitrate analysis, although low amounts of NO_x_-N were observed in most cases, in soils with 1.0% of both untreated and treated feathers, extremely high nitrate values were observed in all checkpoints, obtaining values after 82 days of incubation in soil of 910 mg/L and 864 mg/L, respectively. The presence of high amounts of nitrates in the soil due to the high amount of feathers can lead to nitrate leaching or runoff being most probably the reason for the reduced germination and plant yield for high amounts of feathers, as is also reported by other authors [58].

## 4. Conclusions

This work showed the potential of the incorporation of steam explosion-treated chicken feathers (SEFs) into biodegradable polymeric matrices to obtain biocomposites with accelerated disintegration. The study highlights that the addition of SEFs reduces the density of biocomposites and enhances water absorption due to the hydrophilic nature of feathers. Thermal stability was slightly reduced, while mechanical properties showed increased stiffness but decreased tensile strength, indicating that feathers act more as fillers than reinforcements. Importantly, the biocomposites exhibited accelerated disintegration and biodegradation rates in soil conditions, particularly for PHB and PHBV matrices, with SEF contributing to faster initial degradation stages.

Moreover, ecotoxicity tests revealed that low concentrations of SEF in soil could act as slow-release fertilizers, enhancing crop germination and yield, particularly for cress and barley. However, higher feather concentrations impacted negatively on plant growth due to excessive nitrogen levels. This dual effect (biodegradability and nutrient contribution) boosts the potential of these biocomposites as promising materials for agricultural applications. By valorizing poultry feather waste, this approach not only mitigates environmental pollution but also is aligned with circular economy principles, offering a sustainable solution to the agricultural plastic waste management issue while supporting soil health and crop productivity.

## Figures and Tables

**Table 1:** Processing conditions for the prepared biocomposite formulations.

System	SE Content (%)	Temperature (°C)	Speed (rpm)
PHB	-	170	150
PHB + SEF	10	170	150
PBAT	-	165	150
PBAT + SEF	10	165	150
PHBV	-	165	150
PHBV + SEF	10	165	150
PLA	-	190	150
PLA + SEF	10	190	150

**Table 2:** Relative density and water absorption capacity of biocomposites.

Sample	Relative Density (g cm^−3^)	Swelling (%)	
		t = 1 h	t = 24 h
PHB	1.179	101 ± 1	103 ± 1
PHB + SEF	1.073	104 ± 1	107 ± 1
PLA	1.139	101 ± 1	102 ± 1
PLA + SEF	1.112	102 ± 1	104 ± 1
PHBV	1.207	102 ± 1	104 ± 1
PHBV + SEF	1.148	103 ± 1	106 ± 1
PBAT	1.202	101 ± 1	102 ± 1
PBAT + SEF	1.156	107 ± 2	109 ± 1

**Table 3:** Mechanical properties of the prepared biocomposites and pristine materials.

Sample	Young Modulus (MPa)	Tensile Strength (MPa)	Strain at Break (%)
PHB	2714 ± 156	29 ± 3	3 ± 1
PHB + SEF	2874 ± 75	26 ± 4	2 ± 1
PLA	2516 ± 178	65 ± 3	6 ± 1
PLA + SEF	2541 ± 226	40 ± 3	3 ± 1
PHBV	2231 ± 153	29 ± 2	4 ± 1
PHBV + SEF	3002 ± 101	25 ± 2	2 ± 1
PBAT	119 ± 7	18 ± 1	257 ± 7
PBAT + SEF	200 ± 21	8 ± 1	139 ± 12

**Table 4:** Disintegration behaviour of the prepared biocomposites and pristine materials.

Sample	Disintegration Behaviour After 46 Weeks	Comparison (Week 0 vs. Week 40)
PHB	Pieces of varying size	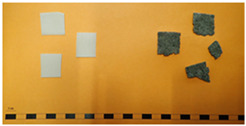
PHB + SEF	100% Disintegration in week 26	-
PLA	All samples remain intact	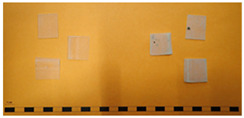
PLA + SEF	All samples remain intact	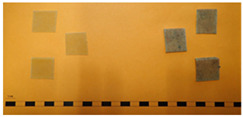
PHBV	100% Disintegration in week 24	-
PHBV + SEF	100% Disintegration in week 26	-
PBAT	Small pieces of varying size	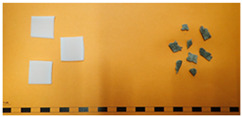
PBAT + SEF	100% Disintegration in week 46	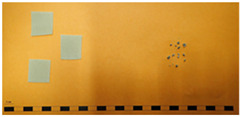

**Table 5:** Germination rate and weight of cress and barley crops in soils containing different amounts of feathers.

Sample	Feather Content (%)	Nitrogen Content (g N/kg soil)	Cress		Barley	
			Germination Rate (%)	Fresh Weight Yield (g)	Germination Rate (%)	Fresh Weight Yield (g)
Blank soil with nutrient	-	-	96 ± 2	7.7 ± 0.6	97 ± 1	13 ± 0
Blank soil without nutrient	-	-	93 ± 2	7.5 ± 0.6	98 ± 0	10 ± 0
S1_SEF	1	1.52	66 ± 7	1.5 ± 0.1	83 ± 3	3 ± 1
S1_RF	1	1.46	93 ± 5	3.0 ± 0.2	88 ± 1	5 ± 1
S0.1_SEF	0.1	0.15	96 ± 3	7.8 ± 0.7	97 ± 2	12 ± 0
S0.1_RF	0.1	0.15	97 ± 1	8.0 ± 0.5	96 ± 2	12 ± 1
S0.05_SEF	0.05	0.08	96 ± 3	7.6 ± 0.7	96 ± 1	11 ± 1
S0.05_RF	0.05	0.08	98 ± 2	8.4 ± 0.4	95 ± 1	11 ± 0

## Data Availability

The original contributions presented in this study are included in the article/Supplementary Material. Further inquiries can be directed to the corresponding author.

## Supplementary Materials

The following supporting information can be downloaded at: https://www.mdpi.com/article/10.3390/polym17070910/s1, Figure S1. Water absorption curves of the prepared biocomposites and matrices; Figure S2. FE-SEM images of PHB, PBAT and PHBV composites containing SEF at different checkpoints during disintegration test; Table S1. Biodegradation in soil test results for the prepared biocomposite with SEF.
