# Supplementary material for: Impact of Steam-Exploded Feather Incorporation on the Biodegradation Performance of Renewable Biocomposites
Source: Polymers (Basel). 2025 Mar 28;17(7):910. doi: 10.3390/polym17070910 (PMC11991220; doi:10.3390/polym17070910)
Supplement: Supplementary file 1 [file polymers-17-00910-s001.zip › polymers-3524487-supplementary.pdf]

Supplementary material

Biodegradable biocomposites from Chicken Feathers: A Circular Economy Approach

Julen Vadillo[1,*], Sarah Montes[1], Hans-Jürgen Grande[1,2], Eveline Beeckman[3], Steven Verstichel[3], Jonna Almqvist[4]

[1] CIDETEC, Basque Research and Technology Alliance (BRTA), Paseo Miramón, 196, 20014 Donostia-San Sebastian, Spain

[2] University of the Basque Country (UPV/EHU), Advanced Polymers and Materials: Physics, Chemistry and Technology Department, Avda. Tolosa 72, 20018, Donostia-San Sebastian, Spain

[3] Normec OWS, Pantserschipstraat 163, 9000 Gent, Belgium

[4] RISE Research Institutes of Sweden, Department of Biorefinery and Energy, S-892 50 Örnsköldsvik, Sweden

** Corresponding author: jvadillo@cidetec.es*

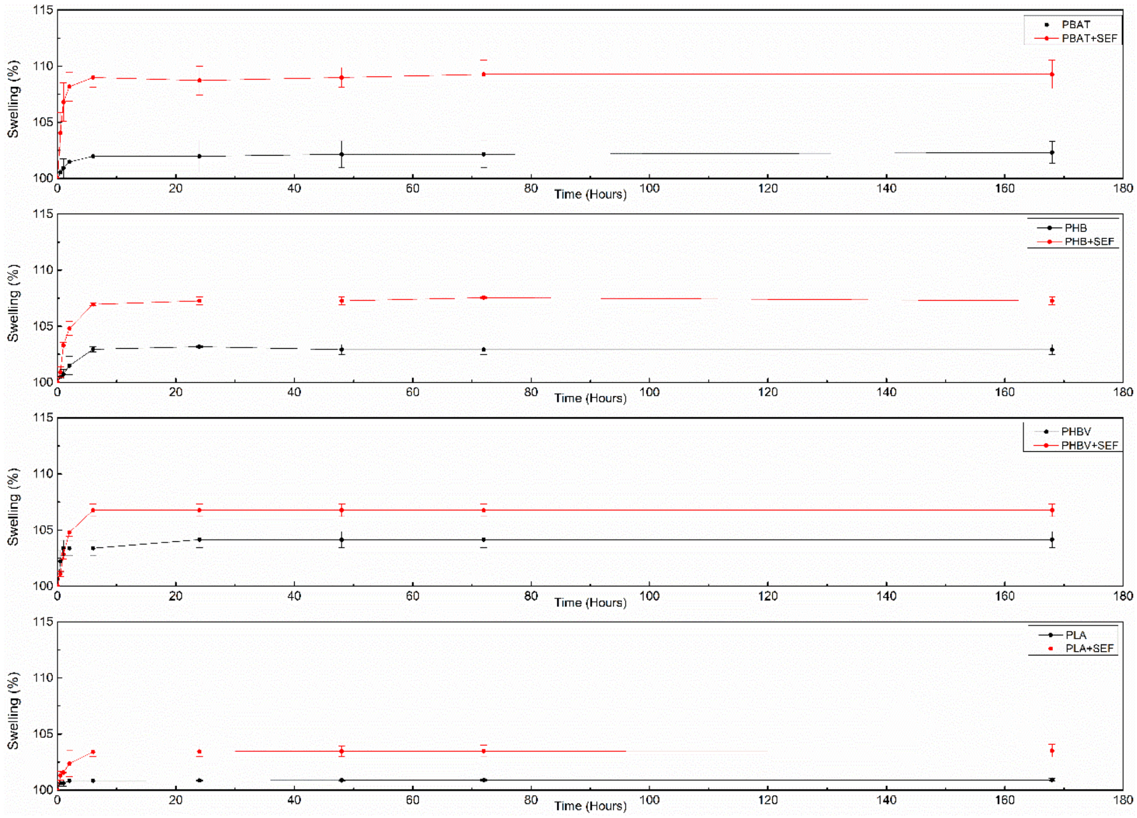

Figure S1 Water absorption curves of the prepared biocomposites and matrices

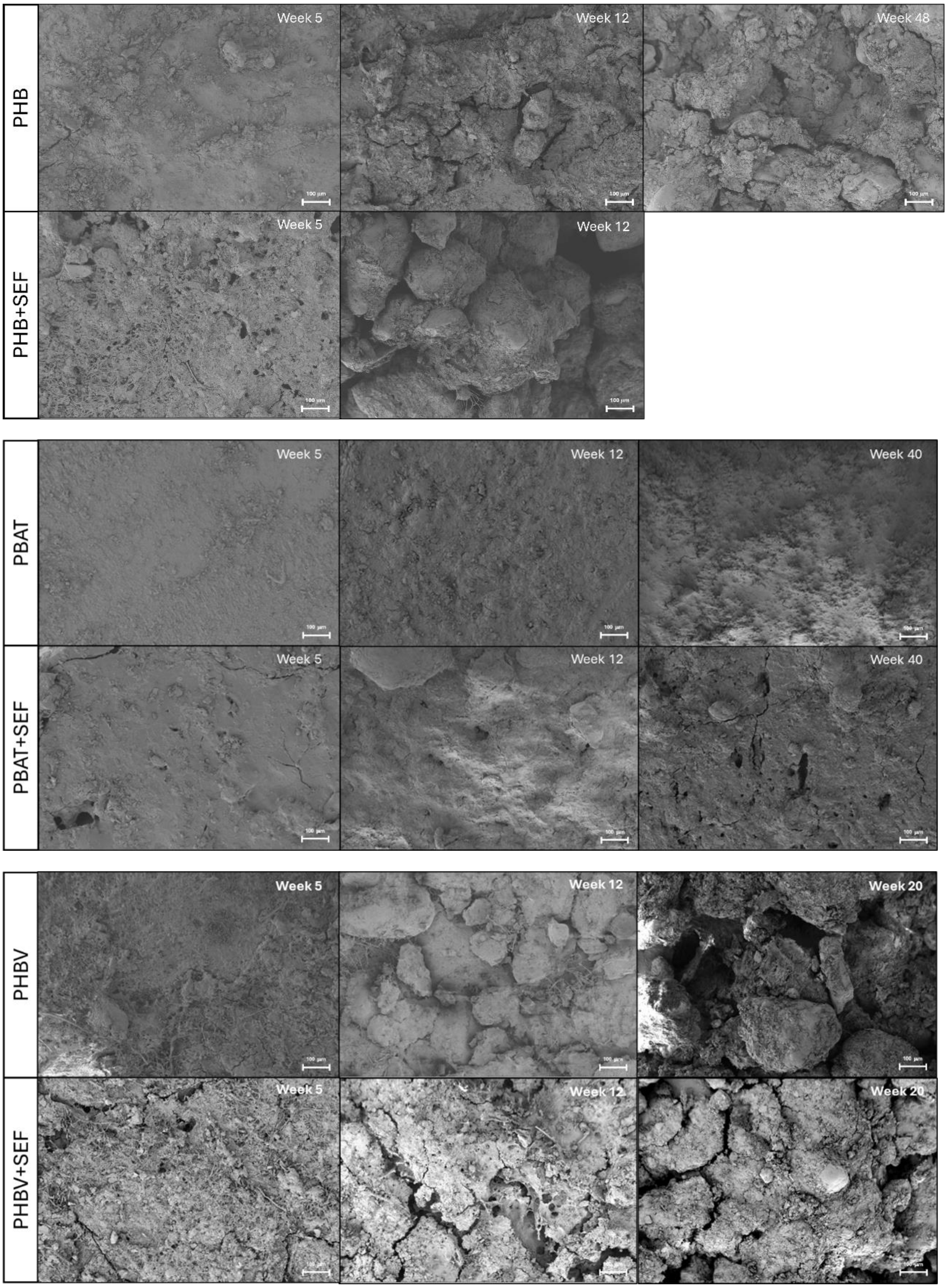

Figure S2 FE-SEM images of PHB, PBAT and PHBV composites containing SEF at different checkpoints during disintegration test

Table S1 Biodegradation in soil test results for the prepared biocomposite with SEF

Test Series	TOC (%)	Net CO_2 production (mg/g test item)	Biodegradation (%)		
			29 days	270 days	
			Average	Average	Relative
Cellulose	42.4	1307	51.8±0.1	84±2	100
PHB	55.6	1746	2.8±1.2	86±1	102
PHB+SEF	54.6	1683	48.2±2.3	84±1	100
PLA	50.4	10	0.1±0.1	0.5±0.3	0.6
PLA+SEF	50.1	287	7.6±2.0	15.6±14.7	18.5
PHBV	56.1	1708	9.8±1.6	83.0±0.1	98.8
PHBV+SEF	55.0	1678	46.4±0.7	83.2±0.2	99.0
PBAT	63.2	479	0.8±0.3	20.7±0.1	24.5
PBAT+SEF	60.6	340	10.1±0.4	15.3±0.7	18.2
